# Progesterone differentially affects the transcriptomic profiles of cow endometrial cell types

**DOI:** 10.1186/s12864-022-08323-z

**Published:** 2022-01-27

**Authors:** Gonçalo Pereira, Yongzhi Guo, Elisabete Silva, Claudia Bevilacqua, Gilles Charpigny, Luís Lopes-da-Costa, Patrice Humblot

**Affiliations:** 1 CIISA-Centro de Investigação Interdisciplinar em Sanidade Animal, Faculdade de Medicina Veterinária, Universidade de Lisboa, Avenida da Universidade Técnica, 1300-477 Lisbon, Portugal; 2 Department of Clinical Sciences, Swedish University of Agricultural Sciences, SLU, PO Box 7054, 750 07 Uppsala, Sweden; 3 Université Paris-Saclay, INRAE, AgroParisTech, GABI, 78350 Jouy-en-Josas, France; 4 Université Paris-Saclay, INRAE, ENVA, BREED, 78350 Jouy-en-Josas, France

**Keywords:** Transcriptome, Progesterone, Endometrium, LCM, RNA-seq

## Abstract

**Background:**

The endometrium is a heterogeneous tissue composed of luminal epithelial (LE), glandular epithelial (GE), and stromal cells (ST), experiencing progesterone regulated dynamic changes during the estrous cycle. In the cow, this regulation at the transcriptomic level was only evaluated in the whole tissue. This study describes specific gene expression in the three types of cells isolated from endometrial biopsies following laser capture microdissection and the transcriptome changes induced by progesterone in GE and ST cells.

**Results:**

Endometrial LE, GE, and ST cells show specific transcriptomic profiles. Most of the differentially expressed genes (DEGs) in response to progesterone are cell type-specific (96%). Genes involved in cell cycle and nuclear division are under-expressed in the presence of progesterone in GE, highlighting the anti-proliferative action of progesterone in epithelial cells. Elevated progesterone concentrations are also associated with the under-expression of estrogen receptor 1 (*ESR1*) in GE and oxytocin receptor (*OXTR*) in GE and ST cells. In ST cells, transcription factors such as *SOX17* and *FOXA2,* known to regulate uterine epithelial-stromal cross-talk conveying to endometrial receptivity, are over-expressed under progesterone influence.

**Conclusions:**

The results from this study show that progesterone regulates endometrial function in a cell type-specific way, which is independent of the expression of its main receptor PGR. These novel insights into uterine physiology present the cell compartment as the physiological unit rather than the whole tissue.

**Supplementary Information:**

The online version contains supplementary material available at 10.1186/s12864-022-08323-z.

## Background

The bovine endometrium is composed of different cell compartments, comprising luminal epithelial (LE), glandular epithelial (GE), and stromal cells (ST), which is submitted to intense tissue remodelling during the estrous cycle, embryo implantation and puerperal involution [1, 2]. Progesterone (P4) released by the corpus luteum (CL) plays a key role regulating endometrial function and remodelling [3, 4]. At the transcriptomic level, this P4 regulation has been evaluated so far, only from the whole endometrial tissue, following luteolysis [5], comparing different oestrus cycle stages [6] or status (diestrus versus anoestrus) and type of ovulation (single versus multiple ovulation) [7]. However, as in other heterogeneous tissues, quantification of gene expression from the whole endometrium may not reflect the specific transcription of the cell compartments. Laser capture microdissection (LCM) emerged as a research tool to isolate cell populations for molecular analyses [8]. This method has been used to study the interactions between the endometrial epithelial and stromal compartments with seminal plasma in a murine model [9] and the conceptus induced regulation of endometrial function in the porcine [10], ovine [11] and equine [12] species. In the cow, recent studies based on this approach described the specific molecular signatures of endometrial stromal, glandular and luminal epithelial cells, as well as the effect of negative energy balance on the transcriptomic profiles of endometrial compartments in the mid-luteal phase [13, 14]. The above experiments revealed that the different endometrial cell types show distinct transcription patterns. The main objective of this study was to evaluate the effects of P4 on the transcription patterns of the three main bovine endometrial cell types, which, to our knowledge, have not been documented so far.

## Results

This study was performed initially while considering the main effects of cell type, P4 concentrations, and their interaction, on the transcriptomic profile of endometrial cells of postpartum cows.

Due to the fact that only one sample from LE cells was associated with high progesterone, transcriptomic changes induced by P4 and their interaction with cell type were studied only from GE and ST cells. From the full list of DEGs (GE plus ST cells), where the main effects was significant (FDR adjusted *P*-value ≤0.05), the interaction was only significant for 1% of these (6 / 591). Furthermore, for most of these genes, significance was mainly associated with a low level of expression. For three genes, the interaction resulted from progesterone being associated with an effect in one cell type but not in the other cell type. For three other genes, (*ERP27*, *GK* and *ENSBTAG00000003408*) the significant interaction resulted from opposite effects of P4 in GE and ST cells. Due to the very low number of genes for which a significant interaction was detected, the results presented and discussed below address principally the main effects of cell type and P4 concentrations.

### Overall gene expression and differentially expressed genes between the three endometrial cell types

**Fig. 1:**
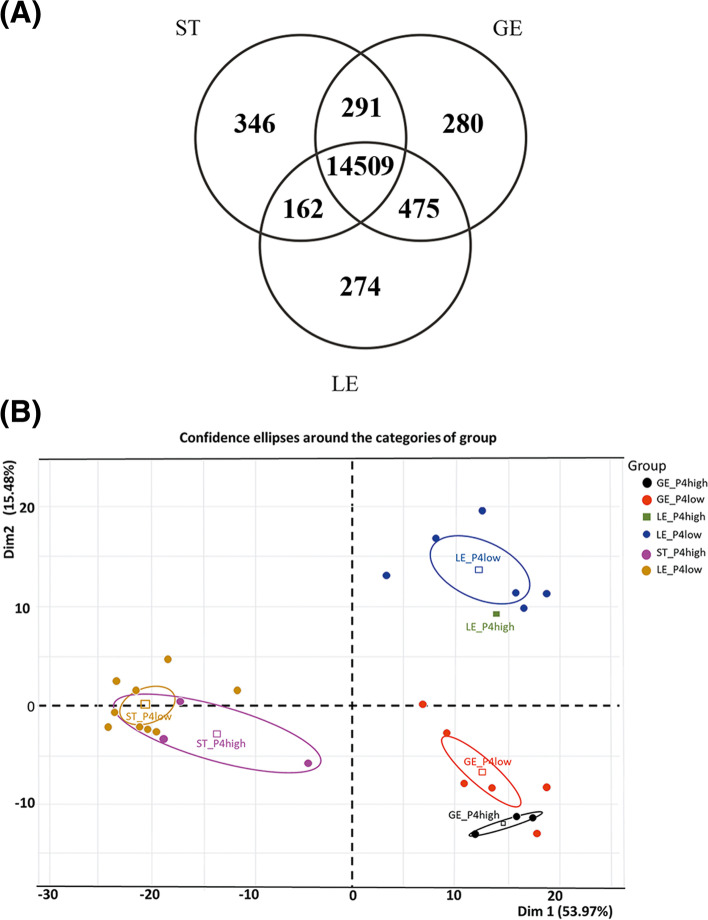
Transcriptome of endometrial cell types and effect of different progesterone concentrations of cows from which samples were issued. **A** Venn diagram with genes expressed (> 10 transcripts per million (TPM)) in endometrial cells (stromal cells (ST), glandular epithelium (GE), and luminal epithelium (LE) (numbers of identified genes are indicated). **B** Principal component analysis (PCA) of cell types (ST, GE and LE) among cow groups (High progesterone, P4high and low progesterone, P4low)

The total number of genes with more than 10 transcripts per million (TPM) was 15,420, 15,555, and 15,308 for LE, GE, and ST cells, respectively. From these, 274 (1.78%) were LE specific, 280 (1.80%) were GE specific and 346 (2.26%) were ST specific (Fig. 1A). Among genes specifically transcribed by LE cells, *TM4SF4*, *C29H11orf86*, *CSF2*, *CAPN14*, *SERPINB10*, *CA1*, *SLC5A5*, *SLC6A12*, and two uncharacterized genes (*ENSBTAG00000036102*, *ENSBTAG00000055111*) had the highest level of average transcription. Among GE-specific genes, *BTG4*, *IBSP*, *TMEM212*, *C4BPA*, *DPP6*, *ANKS4B*, *PROC*, *CFAP58*, *LDLRAD1* and *GAS2L2* were the most transcribed, whereas, among the ST-specific genes, the most transcribed were *CHRNA2*, *KLK5*, *SST*, *DMRT2*, *GABRA4*, *NFASC*, *CDH9*, *DLK1*, and two uncharacterized genes (*ENSBTAG00000054090*, *ENSBTAG00000045630*). The full lists of genes specifically transcribed by each cell type with respective transcription levels are provided in Supplementary File 1.

Although a very high proportion of genes was expressed over 10 TPM in all three cell types (14,509/16337; 88.8%; Fig. 1A), their expression level was not the same. This is shown by the PCA analysis revealing a clear separation of the samples from the three cell types. The two first dimensions explain 70% of the variability (Fig. 1B), the first allowing the distinction of epithelial from stromal cells, whereas the second differentiates GE from LE cells. The genes which explain the most differences in the first dimension (levels of expression the most associated with GE and LE cells) were *TMEM125*, *ESRP1*, *ELMO3*, *KDF1*, *C3H1orf210*, *RHPN2*, *AP1M2*, *CYB561*, *TJP3*, and *SLC44A4*, whereas these genes were *WT1*, *SFRP1*, *TAGLN*, *HOXA10*, *ACTA2*, *C15H11orf96*, *CCN3*, *CNN1*, *TPM2*, and *STRA6* for ST cells. When analyzing differences between GE and LE cells (second dimension), *PPP1R1B*, *MYOC*, *AGT*, *MGP*, *CPN1*, *SNAP91*, *MPTX*, *PRSS35*, *CYBRD1* and *CCDC146* were the genes which were the most specific of GE cells, whereas *BCHE*, *SMOC2*, *GM2A*, *DES*, *ENPP5*, *TNMD*, *ZBTB16*, *CHODL*, *BPIFB1* and *PDZK1* were the most associated with LE cells. The lists of genes most correlated (*P* < 0.01) with each dimension are provided in Supplementary File 2.

The DESeq2 analysis revealed 4045 DEGs between GE and LE cells, 7974 between GE and ST cells, and 7839 between LE and ST cells.

### Gene ontology enrichment analysis of cell-specific genes

Enriched GO terms in each cell-specific genes list were visualized using the REVIGO algorithm to reduce term redundancy and identified 64, 30, and 79 GO terms clusters in LE, GE, and ST cells, respectively (Supplementary Fig. 1). The most significant over-represented terms in LE cells included transport processes, immune system process, cytokine production and regulation, response to stimulus and cell surface receptor signalling, whereas for GE cells, these terms included cilium movement, regulation of triglyceride biosynthetic process, regulation of peptide secretion and transport, regulation of glucose transmembrane transport, and complement activation. For ST cells, the most significant over-represented terms included signalling, cell communication, multiple developmental and biological regulation processes. The complete list of over-represented GO terms is provided in Supplementary File 3.

### Differentially expressed genes between elevated and low progesterone cows

**Fig. 2:**
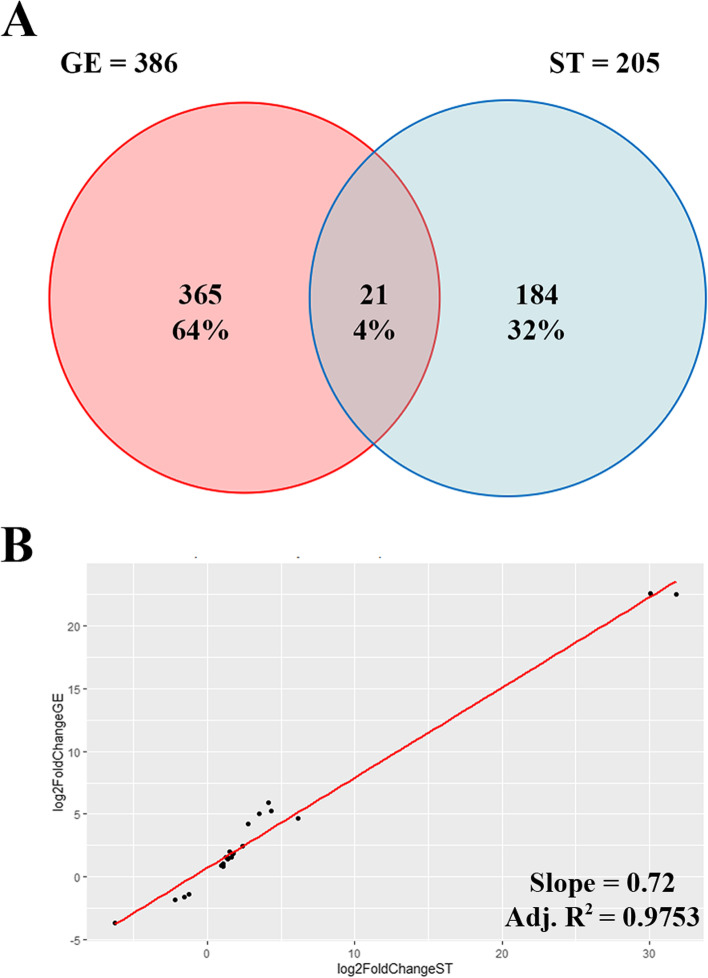
**A** Venn diagram from differentially expressed genes (DEGs) between samples issued from cows of the high and low progesterone groups in glandular epithelial (GE) and stromal (ST) endometrial cells. (Numbers and percentage of DEGs are indicated). B) Linear regression analysis of the fold changes of the 21 common DEGs from ST (x-axis) and GE (y-axis) cells

**Fig. 3:**
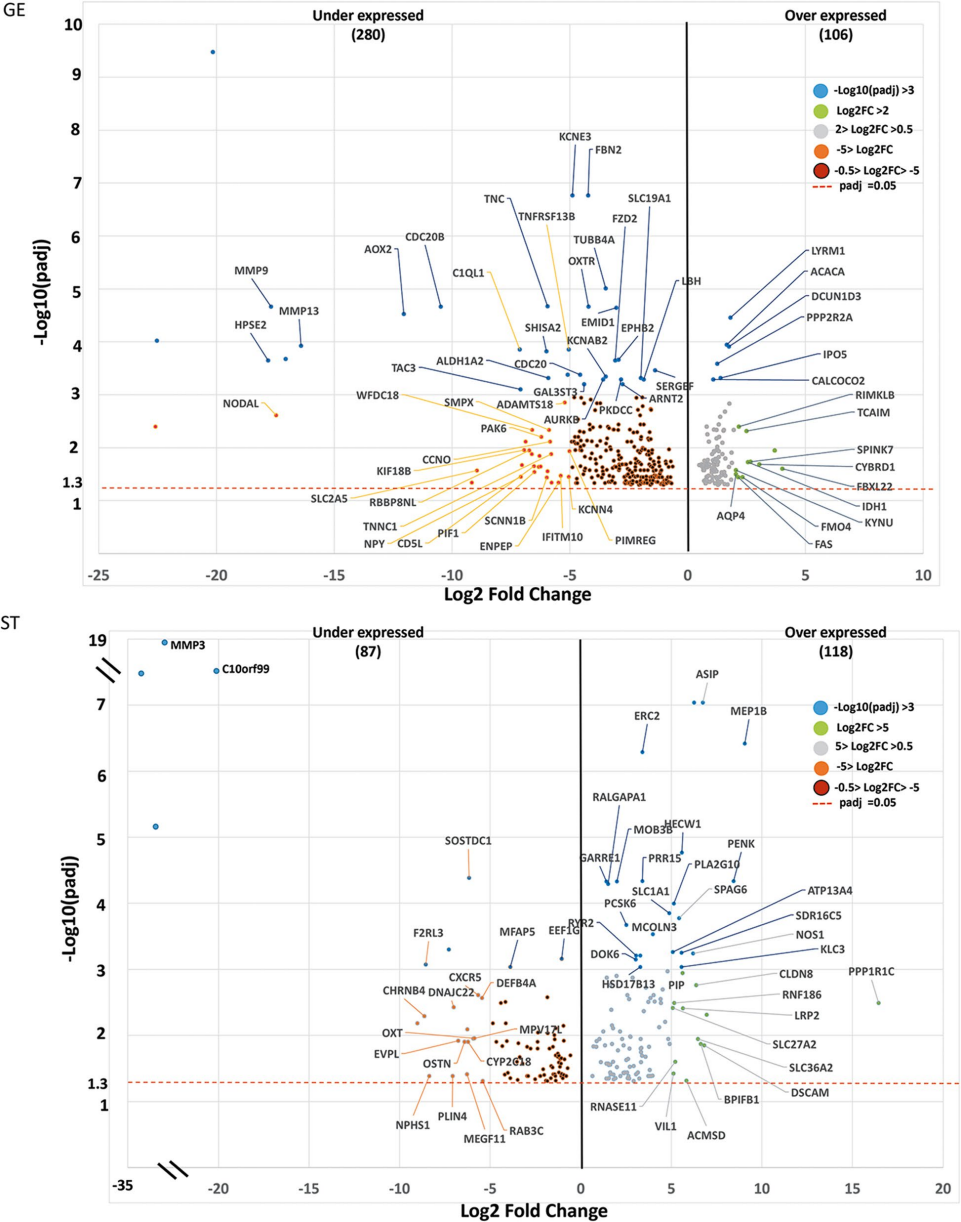
Volcano plots with the distribution of differentially expressed genes between high and low progesterone cows for glandular epithelial (GE) and stromal (ST) endometrial cells

Following microdissection, only 1 high RNA quality LE sample collected from cows with high progesterone concentrations was available. Therefore, differences in the transcriptomic profiles in response to progesterone will be presented and further discussed here from lists of DEGs obtained for GE and ST cells (Supplementary file 4). However, given its potential interest for future studies, the list of putative DEGs from LE samples is also present in Supplementary file 4. The number of DEGs found between samples collected from cows with elevated or low P4 concentrations were 386 and 205 in GE and ST cells, respectively. From these DEGs, 365 (95%) and 184 (90%) were cell type-specific (Fig. 2A). Twenty one DEGs were common to GE and ST cells (*TNC*, *ADAMTS18*, *P4HA2*, *APEX1*, *PNPLA2*, *SOSTDC1*, *TUBB*, *FBLN7*, *MAPK4*, *EEF1G*, *TNFRSF13B*, *ENSBTAG00000015493*, *B9D1*, *RACK1*, *OXTR*, *RPL8*, *C5AR2*, *ENSBTAG00000050840*, *ENSBTAG00000052405*, *TP53INP1, ENSBTAG00000040367*). When considering the above 21 DEGs, the regression analysis of the log2 fold change in response to progesterone from the two cell types showed a similar effect in the two cell types (regression slope = 0.72; adjusted R-squared 0.975; Fig. 2B). The slope coefficient lower than 1 indicates that the magnitude of response was only slightly higher in ST than in GE cells which is consistent with the average log2 fold change for all DEGs observed in ST and GE cells, 3.81 and 2.95, respectively. Moreover, elevated P4 was most often associated with under-expression of genes in GE (280/386; 73%) and over-expression in ST (118/205; 58%) (Fig. 3).

### Gene ontology enrichment analysis of samples from cows with elevated and low progesterone concentrations

**Fig. 4:**
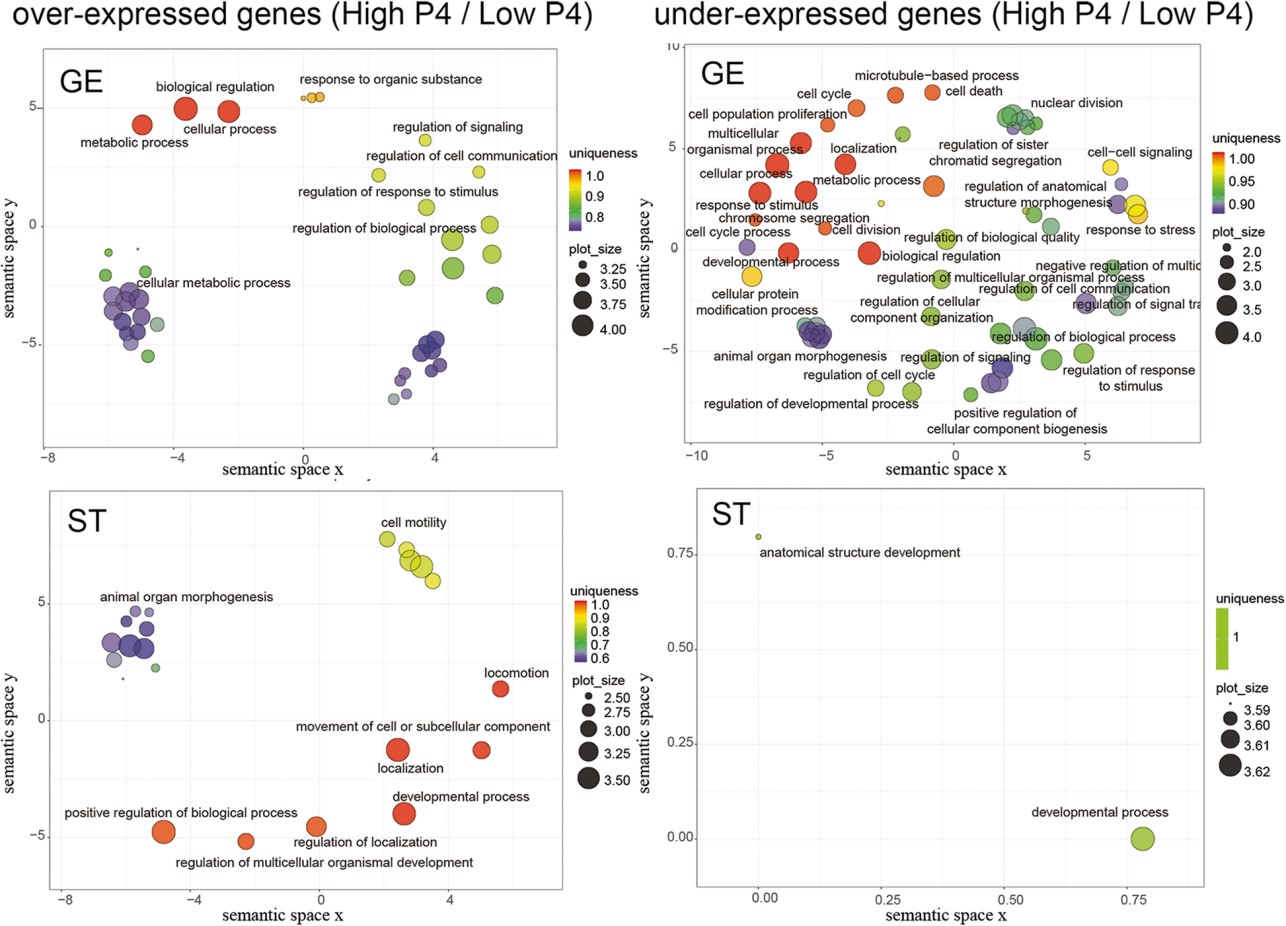
Scatterplot representation of enriched GO terms in semantic space using REVIGO [15], from lists of over-expressed and under-expressed genes in glandular epithelial (GE) and stromal (ST) endometrial cells, between high and low progesterone cows. Circle size represents the frequency of the GO term in the underlying GOA database (bubbles of more general terms are larger) and colour indicates the uniqueness value

Enriched GO terms in the DEGs lists of elevated and low P4 cows are presented in Fig. 4, and the corresponding lists of genes in Supplementary File 5. In GE cells, the over-expressed and under-expressed genes under the effect of P4 relate to 42 and 62 enriched GO terms, respectively. Over-expressed genes are mostly categorised in cellular metabolic processes (GO:0044237) (*n* = 49), response to organic substances (GO:0010033) (*n* = 19) and regulation of diverse biological processes like response to stimulus (GO:0048583) (*n* = 20), cell communication (GO:0010646) (*n* = 18) and signalling (GO:0023051) (n = 18). The under-expressed genes are overrepresented in cell cycle (GO:0007049) (*n* = 31), nuclear division (GO:0000280) (*n* = 14), nuclear chromosome segregation (GO:0098813) (*n* = 14), mitotic cell cycle (GO:0000278) (n = 20), localisation (GO:0051179) (*n* = 58), response to stimulus (GO:0050896) (*n* = 7), response to stress (GO:0006950) (*n* = 36) and cell-cell signalling (GO:0007267) (*n* = 23).

In ST, the analysis revealed 22 and 2 enriched GO terms corresponding to over- and under-expressed genes, respectively. The over-expressed genes relate to cell motility (GO:0048870) (*n* = 14), movement of cell or subcellular component (GO:0006928) (*n* = 18), locomotion (GO:0040011) (*n* = 18), animal organ morphogenesis (GO:0009887) (*n* = 15), localization (GO:0051179) (*n* = 37) and regulation of localization (GO:0032879) (*n* = 18), whereas the under-expressed genes are associated to anatomical structure development (GO:0048856) (*n* = 21) and developmental processes (GO:0032502) (*n* = 22).

### GeneCards and protein-protein interaction (PPI) network analysis

**Fig. 5:**
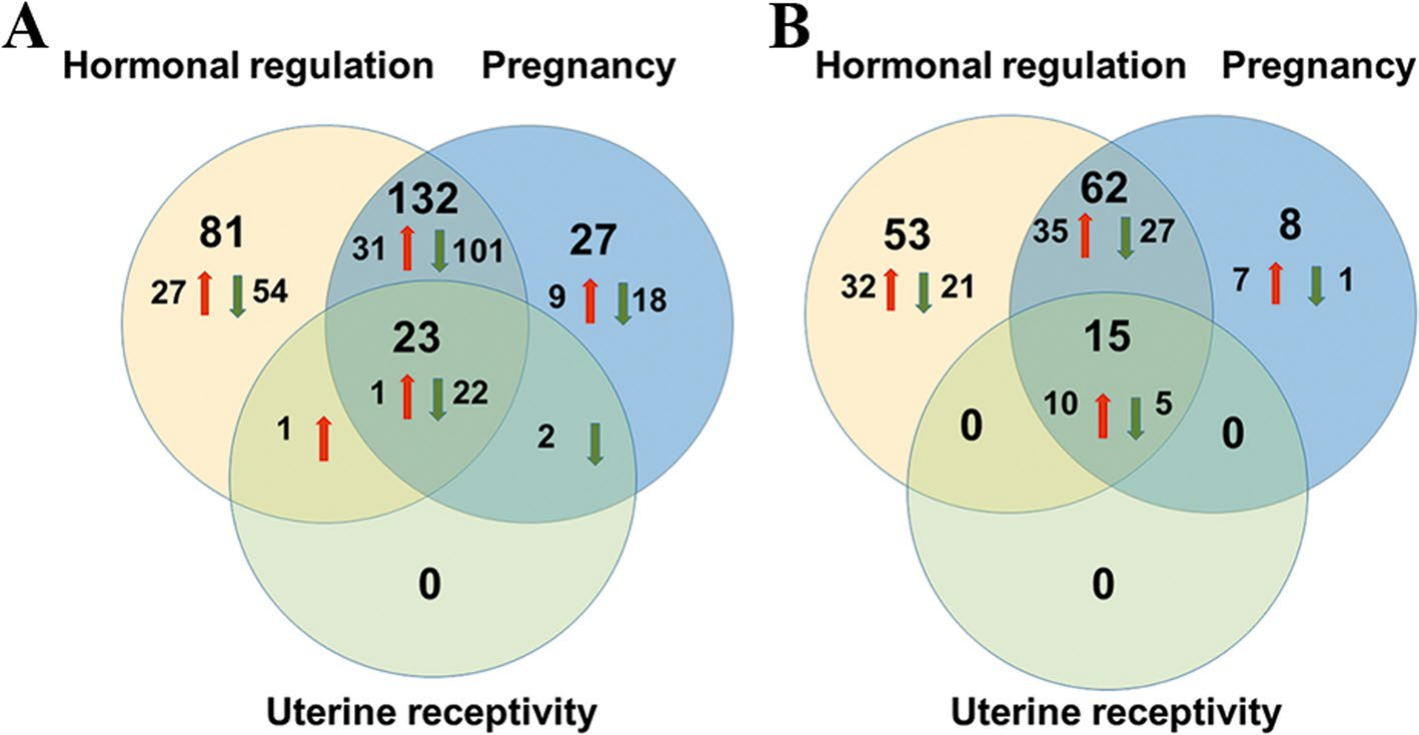
Comparison of differentially expressed genes in glandular epithelial (**A**) and stromal (**B**) cells to GeneCards database. Numbers and sense of variation (arrows) of DEGs participating in hormonal regulation, pregnancy, and uterine receptivity. From the 386 DEGs identified in GE, 237 participate in hormonal regulation, 184 in pregnancy and 26 in uterine receptivity. From the 205 DEGs that emerged in ST, 130 participate in hormonal regulation, 85 in pregnancy and 15 in uterine receptivity

**Fig. 6:**
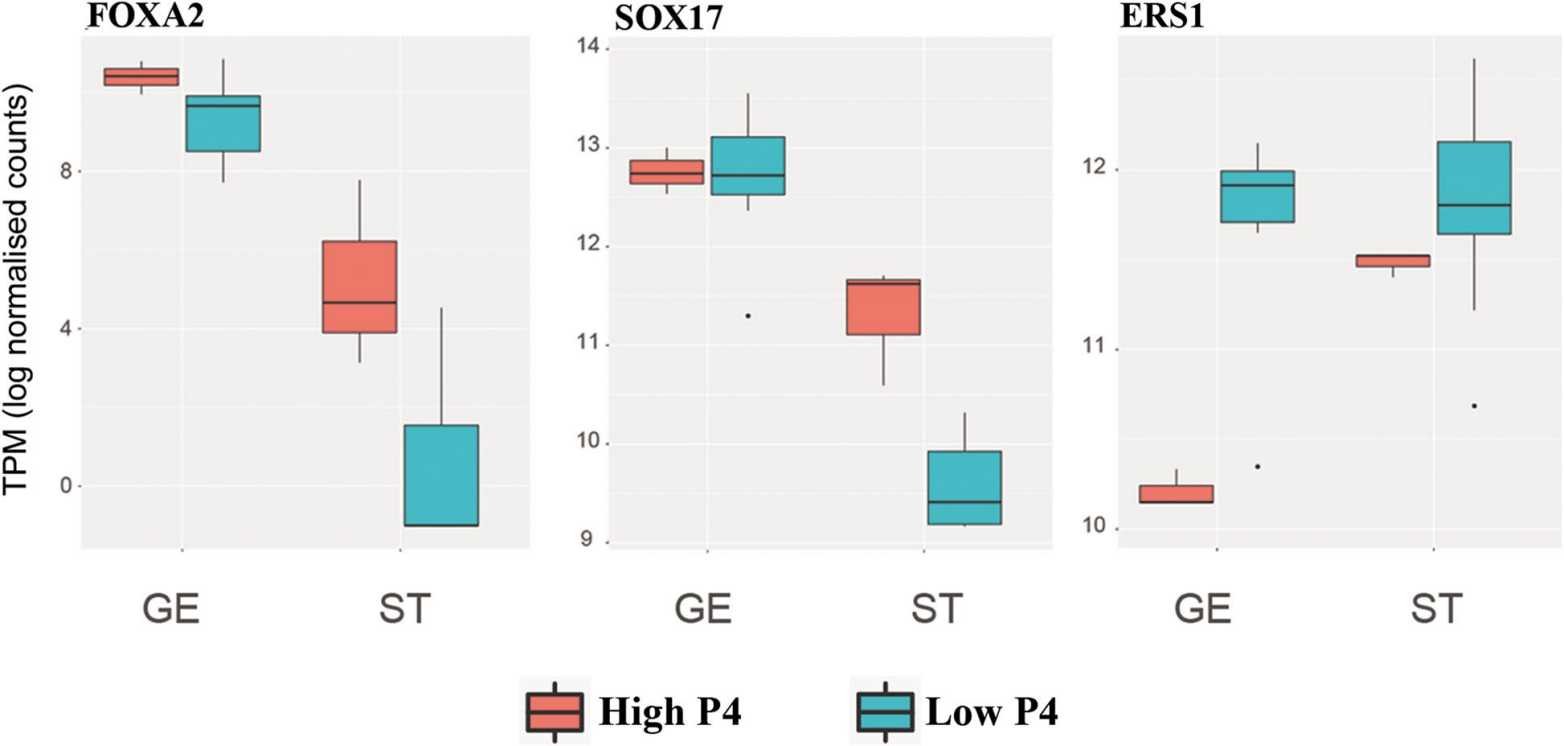
Log normalized transcripts per million (TPM) of *ESR1*, *FOXA2* and *SOX17* genes for glandular epithelial (GE) and stromal (ST) endometrial cells issued from high (red) and low (blue) progesterone cows. Horizontal black lines indicate median; boxes extend from the 25th to the 75th percentile and vertical lines indicate values within 1.5 interquartile range of the 25th and 75th percentiles. Dots indicate outliers

The comparison between the genes of the GeneCards database (http://www.genecards.org) corresponding to “hormonal regulation”, “uterine receptivity” and “pregnancy”, and the DEGs identified in the present study in relation with the presence of P4 is shown in Fig. 5. For GE and ST cells, a very large proportion of DEGs are involved in hormonal regulation (> 60%) and pregnancy (> 40%), and all DEGs participating in uterine receptivity also relates to the above terms. A more thorough analysis of the present DEGs involved in “hormonal regulation”, “pregnancy” and “uterine receptivity” revealed that some of the genes important for these processes are differentially expressed under progesterone in both cell types (Fig. 6). For instance, *ESR1* is under-expressed under the effect of P4 in GE, OXTR is under-expressed under the effect of P4 in GE and ST, and transcription factors such as SOX17 and FOXA2 as well as interferon related genes, which are known to regulate uterine epithelial-stromal cross-talk conveying to endometrial receptivity, are over-expressed under the effect of P4 in ST whereas no change is observed in GE cells.

**Fig. 7:**
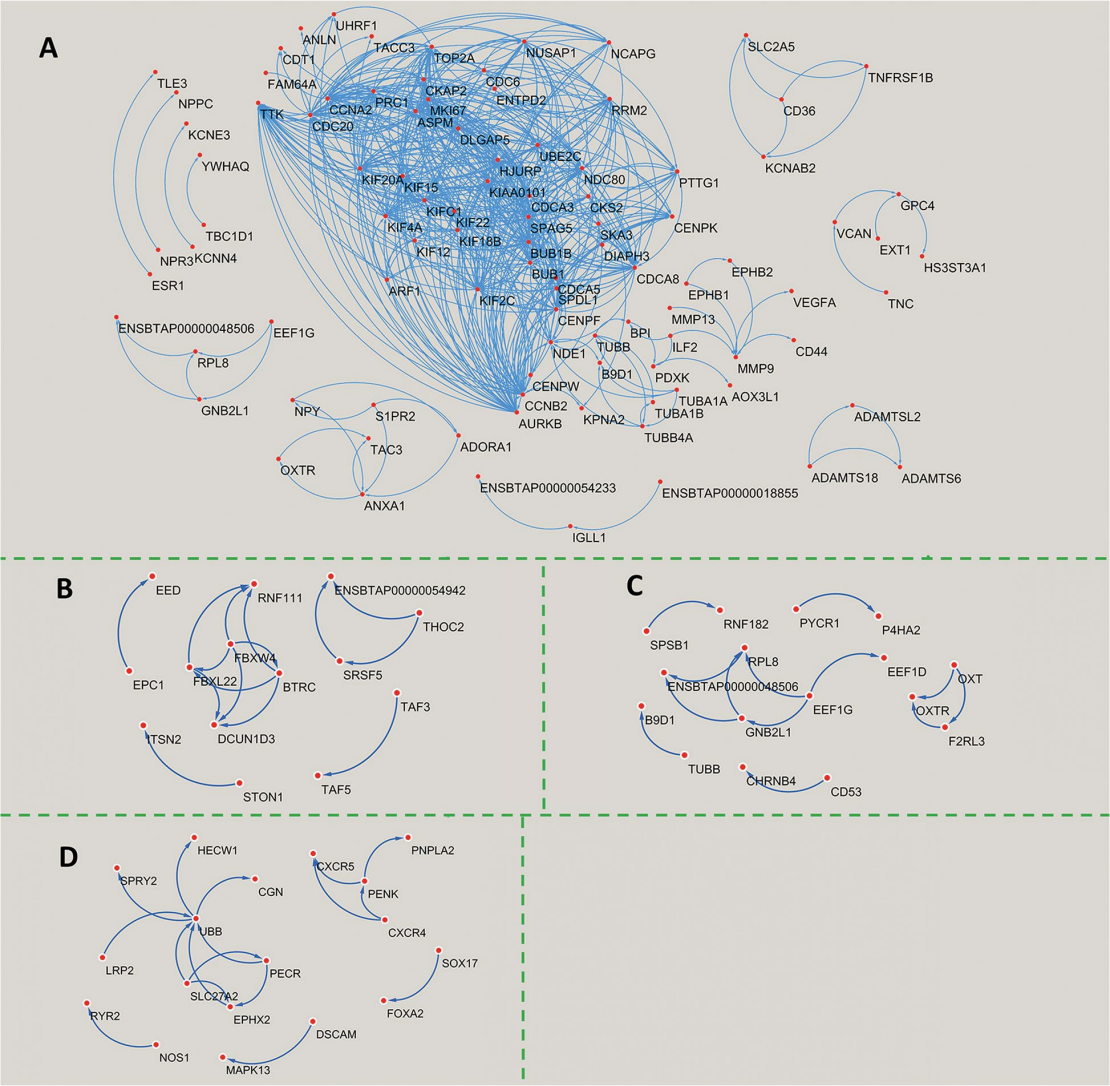
STRING-generated protein-protein networks from differentially expressed genes of glandular (GE), and stromal (ST) endometrial cells between high and low progesterone cows. **A** Under-expressed in GE; **B** Over-expressed in GE; **C** Under-expressed in ST; D) Over-expressed in ST. Arrows pointed to target nodes

In high P4 cows, the STRING-generated protein interaction network obtained from GE DEGs revealed 11 clusters of under-expressed genes including one of a very large size (Fig. 7A) and 5 clusters of over-expressed genes (Fig. 7B), whereas, in ST cells, the analysis revealed 6 clusters of under-expressed (Fig. 7C) and 5 clusters of over-expressed (Fig. 7D) genes.

## Discussion

This study combined LCM to isolate endometrial cell compartments from uterine biopsies and RNAseq to analyze their full transcriptomes, identifying changes induced by P4. This approach provides novel information regarding cell-specific gene expression that remains undetected when analyzing whole tissue samples. This is particularly relevant for epithelial cells for which the cell type’s representability is low, as specific gene expression data is diluted in the average of the whole tissue. Furthermore, as observed here and recently also reported in another model [14], genes may only be differentially expressed in a specific cell type and remain unaffected in the others.

This study confirms the difficulty in capturing samples with good quality RNA from LE. As in Chankeaw et al. [13, 14], despite preparation of a high number of slides from biopsies, the number of sequenced samples was lower for LE than for GE and ST cells.

Of the three different endometrial samples (LE, GE, and ST), ST samples are the most heterogeneous, comprising the *stratum compactum* and *stratum spongiosum*, and including blood vessel and migrating immune cells. By carefully selecting the capture areas, contamination with blood vessel cells was avoided, and contamination with migrating immune cells was minimized by including only healthy cows, as assessed by endometrial cytology and histology. Variation regarding the different strata that compose endometrial ST was addressed by capturing samples from the most superficial layers (*stratum compactum*) as these may induce paracrine interactions with the neighbouring epithelial cells.

The design model here applied revealed strong main effects of cell type and P4 concentrations, but showed lack of significant interaction for most of the genes influenced by progesterone. The fact that *ERP27*, *GK* and *ENSBTAG00000003408)* exhibit opposite changes in expression under P4 in GE and ST cells, would deserve further investigations in relation with endometrial function.

### Transcriptome of endometrial cell types

The PCA from full RNA-seq data corroborates previous work [13] documenting that LE, GE, and ST cells of the bovine endometrium exhibit different molecular signatures. The full description of the characteristics of the transcriptome of the different cell types is beyond the scope of this article (lists are set in Supplementary File 4).

A low percentage (around 2%) of cell type-specific genes were identified, which is in agreement with data from the porcine endometrium [10]. These cell-specific expressed genes encode proteins that putatively support specialized functions of each cell type, which is supported by the overrepresentation of different enriched GO terms in the cell-specific gene lists (Supplementary Fig. 1). Discussion of the compared function of these proteins is beyond the scope of this article, but some relevant examples are summarized below.

LE specific genes encode proteins involved in processes of transport and uptake across epithelial surfaces (solute carrier family 5 member 5, solute carrier family 6 member 12, transmembrane 4 L six family member 4). SLC6 has been shown to be differentially transcribed through different phases of the human oestrus cycle [16] and stimulated by INF-Tau in the cow [17]. These comprise also an enzyme responsible for maintaining acid-base homeostasis (carbonic anhydrase 1), a serine peptidase inhibitor (serpin family B member 10), an embryokine (colony stimulating factor 2), and a member of the calpain family (calpain 14).

Carbonic anhydrase 1 is a member of the large family of zinc metalloenzymes that catalyze the reversible hydration of carbon dioxide and plays a pivotal role maintaining acid-base homeostasis [18] possibly impacting in the endometrium, sperm fertilization capacity, embryo transport, development and implantation [19].

Colony stimulating factor 2 (CSF2) is among the most studied embryokines, being secreted into the bovine uterine lumen [20]. This cytokine is involved in the recruitment, differentiation and function of neutrophils, when secreted by mouse uterine epithelial cells, following stimulation with TLR agonists [21]. CFS2 treatment during the preimplantation period improved the development and survival of bovine embryos [22].

In postpartum dairy cows, LE cells are a primary line of defence against bacteria, and an important component of the innate immune system [2]. This is here evidenced with the over-representation of genes related to immune response and interleukin production, especially interleukin-23 which was shown to be involved in human endometrial immune regulation [23]. Also, the secretory role of LE cells is evidenced by the overrepresentation of processes regarding multiple transport processes (ion, chloride, oxalate, bicarbonate) (Supplementary File 3). Overall, gene data from LE cells highlight their putative specialized functions, such as the regulation of uterine fluid composition, providing favourable microenvironments for sperm and embryos, and the immune response against potential pathogens.

GE specific genes encode proteins involved in cell cycle regulation (BTG anti-proliferation factor 4), adhesion processes (integrin binding sialoprotein), immune response (complement component 4 binding protein alpha, protein C), localized in brush border (ankyrin repeat and sterile alpha motif domain containing 4B, cilia and flagella associated protein 58, low density lipoprotein receptor class A domain containing 1, growth arrest specific 2 like 2) and transmembrane proteins associated with voltage-gated potassium channels (Dipeptidyl peptidase-like protein 6).

The expression of genes encoding proteins related to microvilli adhesion and assembly support that bovine endometrial GE cells form a cluster of tightly packed microvilli, as observed in rat endometrial GE cells [24]. As example, low density lipoprotein receptor class A domain containing 1 is a membrane receptor already identified in GE cells in previous work [13] and also expressed by mature ciliated cells in airway epithelium [25]. Cow endometrial GE cells have specialized functions regarding synthesis, transport and secretion of substances into the uterine lumen [26], here illustrated by the overrepresentation of biological processes of regulation of peptide secretion and cilium movement, the latter believed to be essential for moving secretory products across the surface of GE cells into the uterine lumen [27]. In addition, the role of GE cells in innate and adaptive immune responses is highlighted by the overrepresentation of genes involved in complement activation, as observed in woman’s endometrial GE cells [28]. Complement component 4 binding protein alpha is a protein that controls the activation of the complement cascade and is upregulated in the bovine endometrium following exposure to seminal plasma components, likely involved in the regulation of peri-implantation events [29]. Protein C is a potent anticoagulant and anti-inflammatory molecule which regulates the functions of different epithelial barriers by controlling inflammation [30, 31]. Dipeptidyl peptidase-like protein 6 is a transmembrane protein that binds to voltage-gated potassium channels from the Kv4 family [32], associated with uterine capacity for pregnancy and fertility in beef heifers [33], and relevant for uterine function [34].

The overrepresented processes in ST cells are more numerous and diverse than in GE and LE cells. Most of them relate to regulatory processes, which is consistent with the ST regulatory role exerted on adjacent epithelial cells in co-culture systems [35], or within the cow endometrium [13]. This regulatory role of ST cells also emerges from the overrepresentation of cell communication and signalling processes, which are paramount for the coordination of cellular responses. ST specific genes encode, among others, extracellular proteases (Kallikrein related peptidase 5) and cell adhesion molecules. In women, kallikrein related peptidase 5 is an extracellular protease expressed in endometrial GE cells, suggested to play a role in host defense [36]. This protein is also involved in remodelling and repair of epithelial barriers [37] and able to generate plasmin indicating a role in wound healing [38]. Different collagens were described as substrates for kallikrein-related peptidase 5, hinting a role in extracellular matrix remodelling and cell migration [38].

The specific roles of some of the most correlated genes to each PCA dimension, which were previously found to be involved in endometrial function or associated to pathologies, are also explored below. In the first PCA dimension, which separates stromal from epithelial cells, the most specific ST cells’ gene was the Wilms’ tumor suppressor gene 1 (*WT1*), previously reported as specifically expressed in woman endometrial ST cells [39]. This was also the case of smooth muscle cell markers (*TAGLN*; *ACTA2*; *CNN1*; *TPM2*), also specifically detected in the endometrial stromal compartment of healthy women [40]. Additionally, *STRA6* and *SFRP1*, encoding a receptor for retinol uptake and a soluble modulator of Wnt signalling, respectively, were highly expressed in ST cells, as previously described in woman’s endometrium [41, 42]. The most specific GE and LE genes were those encoding an epithelial splicing regulatory protein (*ESRP1*), which is a regulator of FGFR2 splicing [43], the keratinocyte differentiation factor (*KDF1*), strongly expressed in the dental epithelium of mouse embryos [44], and the Msh homeobox 1 (*MSX1*), with strong nuclear localization in GE and LE cells of fertile woman’s endometrium [45]. In addition, a large set of genes involved in epithelial cell differentiation, epithelium development, and cell adhesion (*CLDN3, SYNE4, LRP2, F2RL1,DLX6, ELF3, SPINT1, PHGDH, OVOL1, TACSTD2, ST14, EHF, MSX1, EPCAM, ST14, KDF1, IRF6, TJP3, SLC44A4, RAB25, DSP, MCOLN3*) were identified, also previously correlated with epithelial cells (GE + LE) in the endometrium of dairy cows [13].

In the second PCA dimension, which separates GE cells from LE cells, the most specific GE cells’ genes were *PPP1RB*, which encodes DARPP-32, a phosphoprotein expressed in ciliary epithelia [46], and *CCDC146*, a ciliated cell marker [47]. Although both LE and GE contain ciliated cells [48], the number of these cells is expected to be lower in LE than in GE, as documented in women [49]. Also as in the human endometrium [50], the angiotensinogen coding gene (*AGT*), was highly expressed in GE cells. A set of genes involved in axonemal dynein complex assembly and cilium movement processes (*CCDC65, DRC1, DRC3, DAW1, CFAP45, DNAH5, DNAH9*) was associated to GE, as previously reported [13].

LE cells were correlated with *SMOC2,* encoding an extracellular glycoprotein recognized as an endometrial cancer stem cell signature [51]. Stem cells were identified in the epithelial and stromal compartments of human endometrium, where they are said to be responsible for its remarkable regenerative capacity [52]. The endometrium of postpartum dairy cows experiences intense tissue remodelling and re-epithelialization, suggesting *SMOC2* as a putative uterine stem cell maker. The desmin coding gene (*DES*) was also specific of LE cells, despite being identified as a smooth muscle cell marker [53]. However, desmin has also been used to distinguish epithelial cells undergoing epithelial-mesenchymal transition (EMT) [54], and there is evidence of EMT participation in endometrial regeneration and re-epithelialization [55, 56]. This indicates desmin expression to be a putative EMT marker in LE cells. *BPIFB1*, a gene encoding an innate defence protein identified in other epithelial barriers, such as human airways [57] was also specific of LE cells.

### Impact of progesterone on the transcription profile of endometrial cell types

For GE and ST cells, the PCA analysis did not identify outliers and individual samples clustered nicely, showing similar gene expression profiles within each P4 group. Overall, data on number of DEGs and overrepresentation of biological processes indicate that the response to elevated P4 was more significant in GE than in ST cells. As documented in the methods section, the average log2 fold change of DEGs in GE and in ST cells (of 2.95 and 3.81, respectively) are associated with a good power to detect differences. A low percentage of common DEGs in GE and ST cells was observed, highlighting the cell type-specific effect of P4 on endometrial gene transcription. Interestingly, the lack of impact of P4 on the expression of P4 receptors (main *PGR* or *PGRMC1, PGRMC2*, *NR2F2*, and *SRD5A2*; data not shown) suggests that the above specific effects are not mediated by the differential binding of P4 to this family of receptors and that other mechanisms should be explored.

As evidenced by the GeneCards analysis, most DEGs in response to progesterone effect participate in hormonal regulation and pregnancy mechanisms and all DEGs involved in uterine receptivity also fall within the two previously mentioned categories. This agrees with the known role of P4 in modulating the transcriptomic profile of the endometrium and modifying the composition of the histotroph for the establishment of uterine receptivity during the pre-implantation period [7, 58–60]. Progesterone is responsible for major changes governing the establishment of uterine receptivity between day 7 and 13 post-estrus, [60]. Changes in endometrial gene expression elicit modifications in the histotroph, including an increase in specific amino acids, glucose, cytokines, and growth factors that support the survival and growth of the conceptus [60]. As reported before, the changes in GE cells include upregulation of meprin A subunit beta (*MEP1B*), a zinc metalloendopeptidase, hypothesized to regulate proteins involved in elongation of the trophectoderm [61]. In addition, this gene was over-expressed in ST cells of cows with elevated P4, suggesting an alternative role in the cleavage of extracellular matrix proteins, as earlier proposed by [61]. Also, the results of our study showing that both *ESR1* and *VEGFA* are under-expressed in GE cells of cows with elevated P4 are consistent with their expression decline during the elongation stage of the conceptus development (from 13 to 20 days post-estrus; review by [59]).

In cows, follicular phase E2 promotes epithelial cell proliferation and endometrial growth, whereas diestral P4 inhibits estrogen-driven epithelial proliferation and promotes differentiation [62, 63]. The anti-proliferative action of P4 in endometrial epithelia of other species [64] is evidenced here in GE cells by the under-expression of genes involved in cell cycle processes. The coordinated and intimate interplay between epithelia and stroma is essential for endometrial response to estrogen (E2) and P4 stimulation (reviewed in humans [65] and mice [66]). In mice, [67] demonstrated that P4 receptors in ST cells are essential for the P4-driven inhibition of epithelial proliferation. There is strong evidence that this inhibition occurs through paracrine interactions and [68] observed that under P4 influence, the transcription factor HAND2 is expressed in stromal cells suppressing the production of several fibroblast growth factors, which are responsible for epithelial proliferation. Moreover, [66] suggested that P4 inhibit Wnt signalling in ST cells, resulting in inhibition of cell cycle progression. In this study, elevated P4 was associated to under-expression of *WNT2* in ST cells. However, ST transcription of *HAND2* was not altered by P4, suggesting a different paracrine loop in bovine endometria.

The over-expression of several genes related with ubiquitin-dependent protein catabolic processes was observed under P4 influence in GE (RNF111, FBXW4, BTRC, DCUND1D3, FBXL22) and ST cells (HECW1, UBB, SPRY2). In women, ubiquitin expression changes along the menstrual cycle and modulates steroid receptor concentrations and endometrial development [69]. In this study, elevated P4 was associated with a strong under-expression of estrogen receptor alpha coding gene (*ESR1*) in GE. This is in accordance with the finding that ESR1 mediating the proliferative role of E2, present its lowest concentrations during the mid-luteal phase [63]. Unlike ESR1, uterine estrogen receptor beta (ESR2) expression is positively associated with increasing P4 concentrations [63]. This association is here supported by the *ESR2* over-expression in ST cells of cows with elevated P4. In addition to its role on ESR1, the present results showing that elevated P4 associated to the under-expression of *OXTR* in GE and ST cells, and *OXT* in ST cells, are in full agreement with former studies describing the role of OXT in luteolysis, as reviewed by [70]. During diestrus, P4 regulates the endometrial expression of oxytocin receptor (OXTR) by suppressing E2 signalling [71], and in pregnant ruminants the conceptus trophoblast produces interferon tau (IFN tau), which downregulates the transcription of *ERS1* and *OXTR* to block the endometrial luteolysis mechanism [72].

In GE, genes encoding tachykinin precursor 3 (*TAC3*) and annexin A1 (*ANXA1*), which are OXTR interacting proteins, were also under-expressed under elevated P4. TAC3 mediates the contractibility of the non-pregnant women uterus [73]. This raises the hypothesis that in cows, P4 can modulate uterine contractibility through the *TAC3* gene. Annexin A1 is a pro-resolving mediator involved in the clearance of apoptotic cells [74, 75]. By down-regulating *ANXA1* expression, elevated P4 induces innate immune response suppression [76]. ANXA2, another member of the Annexin A protein family, was also under-expressed in ST cells of cows with elevated P4. Annexin A2 promotes the formation of phagophores, an essential step in the process of autophagy [77] thus contributing to host immunity during bacterial infection [78]. This result is consistent with work from [1] showing this gene was under-expressed in full tissue biopsies from intercaruncular endometrium of cows at a high P4 stage of the estrus cycle when compared with cows at a low P4 stage of the estrus cycle.

Moreover, genes encoding leukocyte surface antigen (CD53), a tetraspanin involved in regulation of immune cell function [79], and cholinergic receptor nicotinic beta 4 subunit (CHRNB4) were under-expressed under P4 influence in the ST compartment. Since both genes constitute the GO term “neutrophil degranulation” their under-expression under the effect of P4 may also contribute to the higher susceptibility to uterine infections during diestrus in the cow [80].

Elevated P4 was associated with the over-expression of genes encoding proteins involved in endocytosis processes (*ITSN2*, *STON1*) in GE. Endometrial endocytosis occurs in pregnant and non-pregnant cows, mainly during stages at which circulating P4 concentrations are high [81] and during the implantation window in the woman [82]. Although the endometrial role of endocytosis remains unknown, it may be involved in the embryo-endometrium cross-talk during the preimplantation period. In the present study, the overrepresentation of regulation of signalling, cell communication and response to stimulus processes observed in the GE cells of elevated P4 cows are consistent with the above information suggesting that P4 stimulates endocytosis.

As reported before from full tissue biopsies [1], elevated P4 was associated to the over-expression of transcription factors SRY-box transcription factor 17 (*SOX17)* and forkhead box A2 (*FOXA2*) in ST samples from this study. In humans, *FOXA2* is a P4-induced gene involved in transcriptional regulation in endometrial stromal cells [83], and both *SOX17* and *FOXA2* were found to regulate endometrial epithelial-stromal cross-talk related to endometrium receptivity and embryo implantation [84]. As *SOX17* suppresses E2 signalling [84, 85], the network formed with *FOXA2* may represent a mechanism by which transcription of *ESR1* is downregulated in GE of elevated P4 cows (Fig. 6).

In ST, two genes encoding proteins involved in proline metabolism (*P4HA2*, *PYCR1*) were under-expressed under elevated P4. This is consistent with the downregulation of *P4HA2* by progestins in human patients with endometrial hyperplasia [86]. In addition, the knockdown of both *P4HA2* and *PYCR1* reduced cell proliferation of cervical and liver cancer cells, respectively [87, 88]. Taken together, these data suggest that downregulation of *P4HA2* and *PYCR1* may be an additional mechanism by which P4 exerts its endometrial anti-proliferative action in postpartum cows.

Progesterone is also a known inhibitor of cell death, a function supported by the under-expression of tumor necrosis factor receptors when comparing cyclic and non-cyclic cows at 5 weeks postpartum [89]. The results of our study further illustrate this role of progesterone as 4 members of the TNF receptor superfamily were under-expressed under elevated P4 conditions (*TNFRSF13B* in GE and ST cells, *TNFRSF1B* in GE cells, *TNFRSF9* in ST cells, *TNFSF8* in ST cells). In addition, consistently with what was reported before from full tissue biopsies [1], cows with elevated P4 displayed increased gene expression of *EED*, *IDH1*, *SGK3* in GE cells, and *ARHGDIB*, *BCAT1*, *EPHX2*, *LRP2*, *MCOLN3*, *NDRG4*, *PENK*, *PLA2G10* in ST cells. On the contrary, gene expression of *ACP5*, *CLDN10*, *FBLN7*, *GJA1*, *PRDX2*, *TNC*, *TUBA1A*, *TUBA1B*, *TUBB* was decreased in GE cells, and *EEF1G*, *FBLN7*, *MFAP5*, *TNC*, *TUBB* was decreased in ST cells. The present results confirm the former study while allowing a more precise compartment characterization of the effect.

## Conclusion

This study evidences that endometrial cell types have different transcriptome signatures, which are differentially regulated by P4. Under-expression of genes in GE cells by elevated P4 mainly affected cell cycle processes, denoting an anti-proliferative action of P4 in epithelial compartments. In contrast, the elevated P4 regulation of the transcriptomic profile of ST cells is mainly related to the epithelial-stromal cross-talk. Altogether, this study reflects an intricate cell-specific regulation of biological processes in endometrial compartments, which were unnoticed from whole tissue approaches. These results may open paths to understand better the mechanisms regulating endometrial function and their roles with the establishment of pregnancy.

## Methods

### Ethics statement

All animal procedures were conducted by licenced veterinarians, in compliance with the European Union legislation for use of animals for experimental purposes (Directive 2010/63/UE), and the research protocol was approved by the Institutional Animal Care and Use Committee (Reference CEIE n°37/2019).

### Animals

The animal handling and sampling procedures of cows enrolled in this study were published [90]. In brief, high-yielding dairy cows of the Holstein-Friesian breed from a single herd were submitted to blood sampling and genital ultrasonography at 21 ± 0.4 and 44 ± 0.7 days postpartum (DPP) and uterine cytology and uterine biopsy at 44 DPP. The uterine status of all the cows included in this study (*n* = 13) was assessed as healthy, with no clinical signs of endometritis and a percentage of polymorphonuclear leukocytes measured by cytology from 400 cells < 5% at 44 DPP [90, 91]. Additionally, endometrial tissue was confirmed to be healthy as no contamination by immune cells was perceived on histology analysis performed retrospectively. Additionally, at 44 DPP the presence of a CL and plasma P4 concentrations were used to categorize cows in high P4 (*n* = 4) and low P4 (*n* = 9). Ovarian structures as observed by ultrasonography are presented in Table 1. A functional CL was defined as a luteal structure > 23 mm in diameter [92]. Based on heat observation, ovarian ultrasonography and plasma P4 concentrations at 21 and 44 DPP, among the 9 low P4 cows, 4 were still in anoestrus at 44 DPP, and 5 were cyclic at a peri estrus stage of the cycle. From the 4 high P4 cows, 2 had a functional CL arising from the first ovulation postpartum and the other 2 had a functional CL arising from the second ovulation postpartum.

**Table 1:** Cow groups according to clinical characterization and progesterone concentrations

Characteristics	Cows with Low Progesterone concentrations	Cows with High Progesterone concentrations
Number of cows	*n* = 9	*n* = 4
Lactation number^a^	1.8 ± 0.3	1.7 ± 0.4
Results of ovarian ultrasonography		
Non-functional CL	*n* = 4	*n* = 0
Functional CL	*n* = 0	*n* = 4
Progesterone concentrations (ng/mL)^a^	0.5 ± 0.1	5.7 ± 1.4
Range	0.20-0.84	1.8-9.3

^a^mean ± standard error of the mean

### Progesterone assay

Blood was collected by venipuncture of the coccygeal vein into 10 mL dry vacutainers (Becton-Dickinson), allowed to clot and centrifuged (2000 g for 15 min) within 30 min of collection. Serum samples were transferred to the laboratory at 4 °C and then stored at − 20 °C until analysis. Progesterone concentrations were measured by a chemiluminescent immunoassay in an IMMULITE 1000 analyzer (Siemens Healthcare Diagnostics) using a commercial kit (IMMULITE 1000 Progesterone Kit, Siemens Healthcare Diagnostics). The assay’s sensitivity was 0.2 ng/mL, and the inter-assay coefficient of variation was < 10%. The cut-off value used to define cows with high P4 concentrations was 1 ng/mL. Means and range of values for the groups of cow with high and low P4 concentrations are shown in Table 1.

### Endometrial biopsy

Endometrial biopsies were collected with a Kervokian–Younge endometrial biopsy instrument (Alcyon), according to procedures previously described by [90]. The biopsy instrument was guided into the first third of one uterine horn, and an endometrial sample of about 0.5-1 cm^2^ and 3-5 mm thick recovered. The endometrial samples were immediately frozen in dry ice cold isopentane (2-Methylbutane, Sigma Aldrich) for 60 s and embedded in a cryomold with optimal cutting temperature compound (Tissue-Tek OCT Compound, Sakura Finetek). Cryomolds were transferred to the laboratory on dry ice, then kept at − 80 °C until tissue processing.

### Endometrial tissue processing and staining

Serial sections (8 μm thick) were cut from the tissue blocks on a cryostat (Cryotome FSE, Thermo Scientific) set at − 20 °C, mounted on glass slides at 4 °C and immersed for 60 s in 75% ethanol inside the cryostat chamber (− 20 °C). Slides were then stained with Cresyl Violet (1% in 50% ethanol) and dehydrated at room temperature as described [93]. When taken out of the cryostat chamber, the slides were transferred to 75% ethanol for 20 s, stained with 1% Cresyl violet in ethanol (25 s), rinsed successively with 75% ethanol (30 s), 95% ethanol (2 × 1 min), 100% ethanol (2 × 1 min), and finally, pure xylene (M-xylene, Sigma-Aldrich; 2 × 5 min). In order to ensure appropriate dehydration, new bottles of absolute ethanol and pure xylene were opened every day immediately before use. Stained and dehydrated tissue sections were air-dried to remove xylene residues before microdissection.

### Laser capture microdissection and RNA extraction

**Fig. 8:**
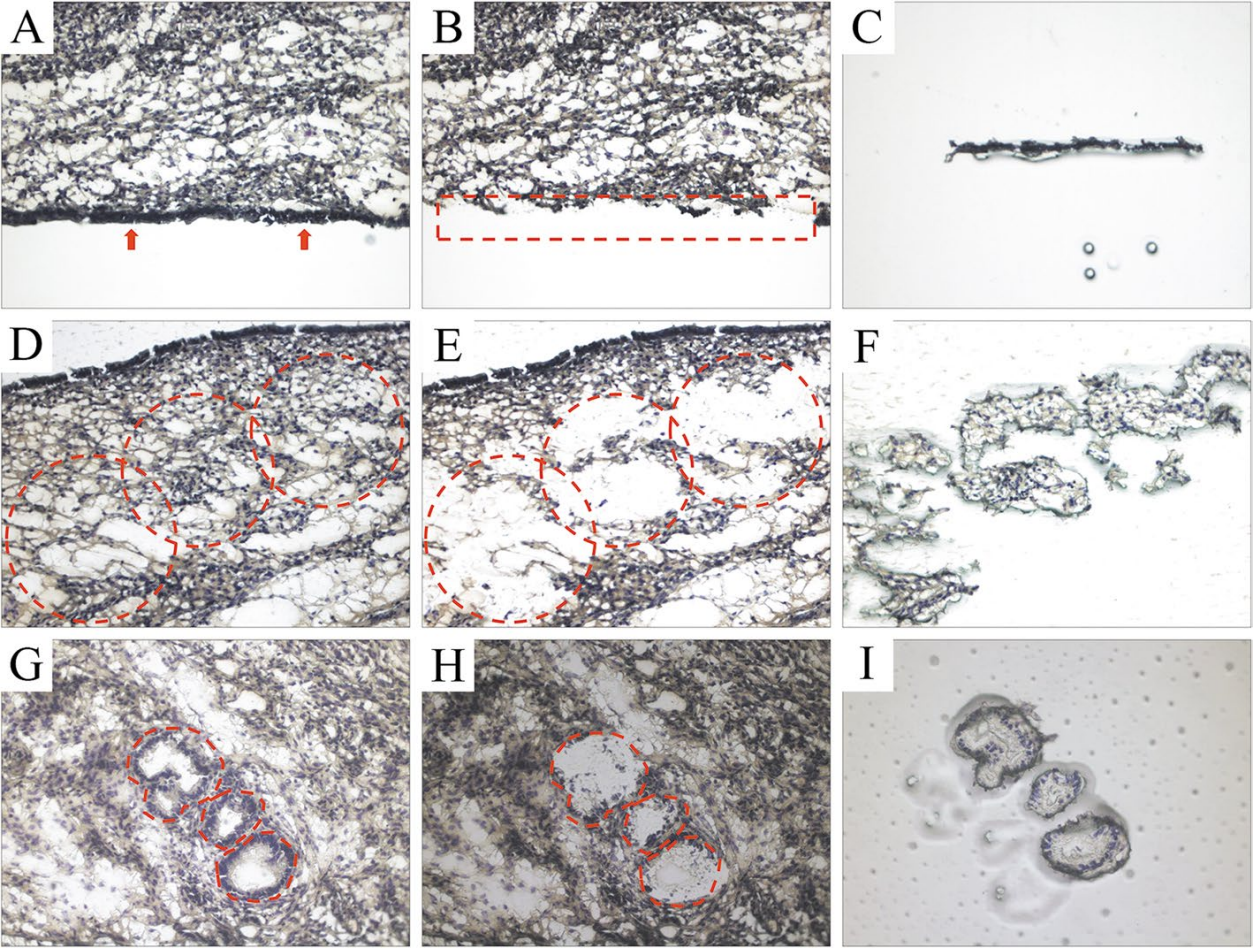
Histologic aspect of endometrial cell types before and after Laser Capture Microdissection (LCM). **A**, arrows pointing to luminal epithelial (LE) cells before LCM; **B**, dashed rectangle highlighting the place where the LE cells were on the slide; **C**, captured LE cells in the LCM plastic caps; **D**, dashed circles highlighting the stromal (ST) cells; **E**, dashed circles highlighting the place where the ST cells were on the slide; **F**, captured ST cells in the LCM plastic caps; **G**, dashed shapes highlighting the glandular epithelial (GE) cells; **H**, dashed shapes highlighting where the GE cells were on the slide; **I**, captured GE cells in the LCM plastic caps. (400× magnification)

The endometrial cell types (LE, GE and ST) were isolated from the whole tissue sections using an ARCTURUS XT™ Laser Capture Microdissection System and software (Applied Biosystems®, Arcturus). Although cells isolated from the ST compartment may comprise a combination of fibroblasts, immune and endothelial cells, ST is hereafter referred to as a “cell type”. A previous report showed that contamination of micro-dissected ST samples by other cell types was negligible [13]. Laser capture was performed either under 20× or 40× magnification and infrared settings (power, duration and intensity) were adjusted for each field of view, to maximize the size of the laser spot without contaminating the sample with undesired cells. Following capture, each LCM plastic cap (CapSure®Macro LCM Caps, Arcturus) was examined at the quality control (QC) station and if necessary, undesired cells were removed from the cap by low power UV laser. For a given tissue section, the full microdissection processing did not last more than 90 min to preserve RNA integrity. Examples of histology slides of each endometrial cell type before and after capture with LCM are presented in Fig. 8. After microdissection, total RNA from LE, GE and ST cells was extracted using the PicoPure™ RNA Isolation Kit (Arcturus) following the manufacturer’s protocol. A DNAse I (Qiagen) treatment step was added according to the protocol and eluted in 15 μl of Elution buffer. The RNA quantity and quality [RNA Integrity Number (RIN)] were assessed with the Agilent Bioanalyzer 2100 system (Agilent Technologies) and the RNA 6000 p Chip Kit. Due to difficulty in harvesting enough RNA with eligible RIN value (≥ 7) for gene expression measurements [93], from the initial 39 samples from 13 cows, only 7 LE, 10 GE, and 12 ST samples were analyzed by RNA sequencing (Table 2).

**Table 2:** Number of samples used for each cell type and cow group with respective mean RIN values (mean ± SEM)

Endometrial cell types	Cow sub-group		RIN^a^
	Low P4	High P4	
Stromal cells	9	3	7.41 (± 0.24)
Glandular epithelial cells	7	3	7.23 (± 0.17)
Luminal epithelial cells	6	1	7.54 (± 0.19)

^a^RNA Integrity Number, values reported as mean ± SEM

### RNA sequencing and data analysis

RNA sequencing libraries from 29 samples were prepared and sequenced on the GenomEast Platform (IGBMC, Cedex, France; http://genomeast.igbmc.fr/). Full-length cDNA was generated from 2.5 ng of total RNA using Clontech SMART-Seq v4 Ultra Low Input RNA Kit for Sequencing (Takara Bio Europe, Ozyme) according to manufacturer’s instructions, with 10 cycles of PCR for cDNA amplification by Seq-Amp polymerase. Then, 600 pg of pre-amplified cDNA was used as input for Tn5 transposon tagmentation using the Nextera XT DNA Library Preparation Kit (Illumina), followed by 12 cycles of library amplification. Following purification with Agencourt AMPure XP beads (Beckman-Coulter), the size and concentration of libraries were assessed by capillary electrophoresis. Sequencing was performed on an Illumina HiSeq 4000 with 100 bp paired-end reads. Image analysis and base calling were performed using RTA 2.7.3 and bcl2fastq 2.17.1.14. The sequencing depth of RNA-seq libraries was in the range of 72 to 83 million reads per sample and all samples had a quality score over 30, meaning that the base call accuracy was 99.9%, in at least 90% of the sequenced bases. Gene level exploratory analysis and differential transcription analysis were performed using the RNAseq workflow described by [94] (update version https://bioconductor.org/help/course-materials/2017/CSAMA/labs/2-tuesday/lab-03-rnaseq/rnaseqGene_CSAMA2017. html). The Salmon method [95] was used to quantify transcript abundance. Tximport method [96] (R package version 1.8.0) was then used to import Salmon’s transcript-level quantifications. The cDNA sequence database for *Bos taurus* was obtained from Ensembl (release-98; Bos_taurus.ARS-UCD1.2.cdna.all.fa) and was used to build a reference index for the bovine transcriptome (see details in [95]). Power analysis was performed using the method described by [97] and compiled in the R package ssizeRNA (version 1.3.2). Calculated at an FDR of 0.05, power was 15, 58, 87% to detect 1.5, 2 and 3 log2 fold change in GE cells, and 24, 63, 87% to detect 1.5, 2 and 3 log2 fold change in ST cells.

### Gene expression analysis

Following quantification of RNA-seq data, transcripts whose average value computed from biological replicates was less than 10 TPM were regarded as biological background noise and were not considered to identify the number of genes specifically expressed by each cell type. Principal component analysis (PCA) was performed with DESeq2 and FactoMineR (R package, version 1.4.1) using the variance stabilizing transformation output files from DESeq2. Venn diagrams were plotted with VennDiagram package (1.6.20). The DESeq2 package (R package, version 1.26.0) was used for the analysis of differential expressed genes (DEGs) with the corresponding statistical methods [94] including tests for differential transcription by use of negative binomial generalized linear models. The following terms were added in the design formula (cell_type + progesterone_group + cell_type: progesterone_group) to test the main effects of endometrial cell types and P4 concentration groups, as well as their interaction with the false discovery rate adjusted *p*-value of 0.05 used for the identification of DEGs. False discovery rate adjustment was performed using the Benjamini and Hochberg method [98]. In all comparisons, ratios for fold change are expressed as mean TPM from cows with elevated P4 / mean TPM from cows with low P4. Cell-specific genes were defined as genes with average TPM ≥ 10 for a given cell population and average TPM < 10 for the other cell populations. Data were deposited in NCBI’s Gene Expression Omnibus and are accessible through GEO Series accession number GSE182932 (https://www.ncbi.nlm.nih.gov/geo/query/acc.cgi?acc=GSE182932).

### Gene Ontology (GO) and pathway enrichment analysis

Significant GO terms of the “Biological Process” domain were found with the GO-TermFinder software [99], summarised with similarity coefficient at low or medium level, and visualized in semantic space by REVIGO (http://revigo.irb.hr/) [15]. When analyzing the lists of specific cell type genes, GO-TermFinder settings were set to *P* < 0.01. The lists of DEGs from the different cell types between elevated and low P4 cows were analyzed with Bonferroni adjustment and FDR settings at *P* < 0.01. Analysis of DEGs possibly involved in “hormonal regulation”, “uterine receptivity”, and “pregnancy” terms was based on GeneCards database (http://www.genecards.org/), as previously described [100].

### Construction of protein-protein interaction (PPIs) networks

The interaction networks among proteins encoded by DEGs from the different cell types between elevated and low P4 cows were constructed with STRING database v11.0 (http://string-db.org) [101]. All PPIs network were generated at a confidence score of 0.9 with “non/query protein only”, and the sources of active interaction were all selected (Textmining, Experiments, Databases, Co-expression, Neighborhood, Gene Fusion, and Co-occurrence). Then the networks were sent to Cytoscape v 3.8.2 and were visualized by yFiles layout algorithms for the Cytoscape app.

## Supplementary Information

**Additional file 1: Supplementary File 1.** TPM cell type. Description: List of Transcripts cell type-specific.

**Additional file 2: Supplementary File 2.** PCA correlated Genes. Description: List of genes correlated with the 2 dimensions of the PCA analysis.

**Additional file 3: Supplementary File 3.** Go terms cell type-specific. Description: List of GO terms over-represented in cell type-specific lists of transcripts.

**Additional file 4: Supplementary File 4.** Lists of DEGs. Description: Lists of genes differently expressed in LE, GE and ST cells from high and low progesterone cows.

**Additional file 5: Supplementary File 5.** GO terms over-represented in DEGs lists. Description: List of GO terms over-represented in lists of genes differently expressed in GE and ST cells from high and low progesterone cows.

**Additional file 6: Supplementary Fig. 1.** Scatterplot representation of enriched GO terms in semantic space using REVIGO (Supek et al. 2011), from lists of cell-specific genes of luminal epithelial (LE), glandular epithelial (GE) and stromal (ST) cells. Circle size represents the frequency of the GO term in the underlying GOA database (bubbles of more general terms are larger) and colour indicates the uniqueness value.

## Abbreviations

CL: Corpus Luteum
DEG: Differentially Expressed Gene
DPP: Days PostPartum
E2: Estrogen
EMT: Epithelial Mesenchymal Transition
ESR1: Estrogen Receptor 1
GE: Glandular Epithelial
GO: Gene Ontology
LCM: Laser Capture Microdissection
LE: Luminal Epithelial
OXTR: Oxytocin Receptor
P4: Progesterone
PCA: Principal Component Analysis
PPI: Protein-Protein Interaction
QC: Quality Control
RIN: RNA Integrity Number
ST: Stroma
TPM: Transcripts Per Million
