# Supplementary material for: Progesterone differentially affects the transcriptomic profiles of cow endometrial cell types
Source: BMC Genomics. 2022 Jan 27;23:82. doi: 10.1186/s12864-022-08323-z (PMC8793221; doi:10.1186/s12864-022-08323-z)
Supplement: Supplementary file 6 — Additional file 6: Supplementary Fig. 1. Scatterplot representation of enriched GO terms in semantic space using REVIGO (Supek et al. 2011), from lists of cell-specific genes of luminal epithelial (LE), glandular epithelial (GE) and stromal (ST) cells. Circle size represents the frequency of the GO term in the underlying GOA database (bubbles of more general terms are larger) and colour indicates the uniqueness value. [file 12864_2022_8323_MOESM6_ESM.pdf]

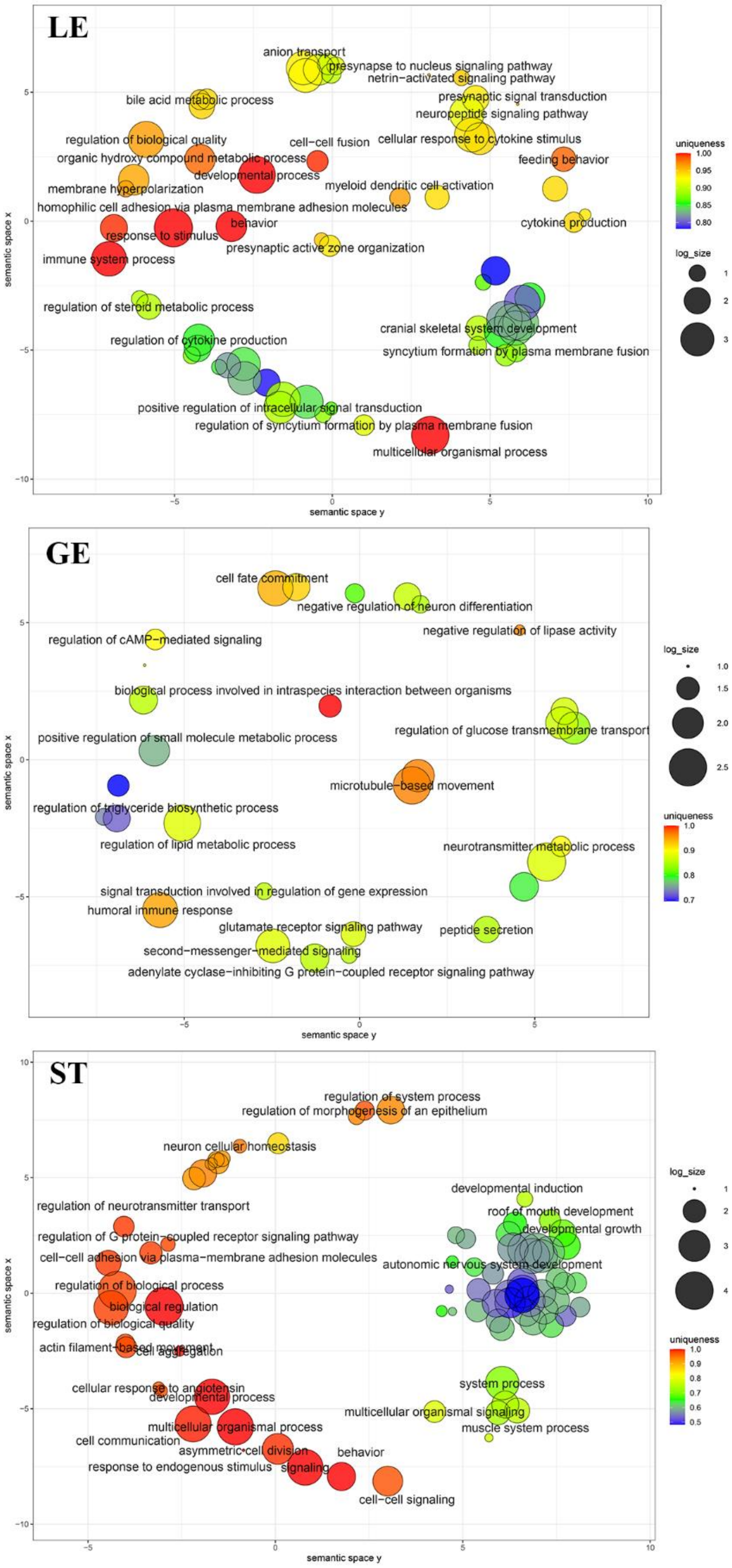

Supplementary Fig. 1. Scatterplot representation of enriched GO terms in semantic space using REVIGO (Supek *et al*. 2011), from lists of cell-specific genes of luminal epithelial (LE), glandular epithelial (GE) and stromal (ST) cells. Circle size represents the frequency of the GO term in the

underlying GOA database (bubbles of more general terms are larger) and colour indicates the uniqueness value.
